# Radiological score, asthma and NSAID‐exacerbated respiratory disease predict relapsing chronic rhinosinusitis

**DOI:** 10.1002/clt2.70043

**Published:** 2025-04-02

**Authors:** Markus Lilja, Anni Koskinen, Sari Hammarèn‐Malmi, Anu Laulajainen‐Hongisto, Jura Numminen, Jyri Myller, Seija Vento, Elina Penttila, Maija Hytönen, Paula Virkkula, Peter W. Hellings, Sven F. Seys, John Lee, Heini Huhtala, Johanna Sahlman, Sanna Toppila‐Salmi

**Affiliations:** ^1^ Department of Allergology Inflammation Center Helsinki University Hospital and University of Helsinki Helsinki Finland; ^2^ Department of Otorhinolaryngology—Head and Neck Surgery Helsinki University Hospital and University of Helsinki Helsinki Finland; ^3^ Department of Otorhinolaryngology Hyvinkää Hospital Hospital District of Helsinki and Uusimaa Hyvinkää Finland; ^4^ Department of Otorhinolaryngology & Head and Neck Surgery Faculty of Medicine and Health Technology Tampere University Hospital Wellbeing Services County of Pirkanmaa Tampere University Tampere Finland; ^5^ Department of Otorhinolaryngology Wellbeing Services County of Päijät‐Häme Päijät‐Häme Central Hospital Lahti Finland; ^6^ Department of Otorhinolaryngology Kuopio University Hospital Wellbeing Services County of North Savo Kuopio Finland; ^7^ Department of Otorhinolaryngology, Head and Neck Surgery UZ Leuven Campus Gasthuisberg Upper Airways Research University of Ghent Leuven Belgium; ^8^ Allergy and Clinical Immunology Research Group Department of Microbiology, Immunology & Transplantation KU Leuven Leuven Belgium; ^9^ Department of Otolaryngology–Head and Neck Surgery St. Michael's Hospital University of Toronto Toronto Ontario Canada; ^10^ The Keenan Research Centre in the Li Ka Shing Knowledge Institute of St. Michael's Hospital Toronto Ontario Canada; ^11^ Faculty of Social Sciences Tampere University Tampere Finland; ^12^ Department of Otorhinolaryngology University of Eastern Finland Kuopio Finland; ^13^ Haartman Institute University of Helsinki Helsinki Finland

**Keywords:** antrochoanal polyp, aspirin exacerbated respiratory disease, aspirin intolerance, ESS, inflammation, nasal polyp, recurrence, revision surgery, sinus surgery, sinusitis

## Abstract

**Objectives:**

The aim was to evaluate the predictive potential of Sinonasal Radiological (SR) and the Lund‐Mackay (LM) score of sinus computed tomography (CT) scans on postoperative relapses of chronic rhinosinusitis (CRS).

**Materials and Methods:**

CRS patients (*n* = 483, 12–80 years) underwent routine sinus CT scans. The SR score was defined by obstructed frontal recess (0 = no, 1 = yes) and visualization of middle and inferior turbinate (0 = anatomy can be easily visualized, 1 = anatomy cannot be easily visualized) on each side (a total of 0–6 points). Associations were analyzed by nonparametric, survival and Cox's proportional hazard models.

**Results:**

Revision endoscopic sinus surgery (ESS) was performed in 133 (28.0%) patients on average (min–max) of 3.2 (0–12) years after performing the sinus CT scans. Of the 408 patients who underwent the baseline ESS, high preoperative SR or LM scores significantly predicted revision ESS (*p* < 0.001) and peroral corticosteroid courses purchased during the follow‐up (*p* = 0.009 and *p* < 0.001, respectively for SR‐ and LM‐scores). In multivariable analysis, both SR score and asthma and/or NSAID exacerbated respiratory disease (N‐ERD) were significantly associated with revision ESS risk (*p* = 0.035, *p* = 0.007, respectively).

**Conclusion:**

LM and SR and a history of asthma or N‐ERD predict CRS relapses, which may help in decision‐making.

## 1 INTRODUCTION

Chronic rhinosinusitis (CRS) is an inflammatory disease of the nose and paranasal sinuses with little knowledge of predictors of its relapses.^1^ The prevalence of CRS is around 5%–12%^2^ and it has a severe impact on quality of life and work productivity. Chronic rhinosinusitis causes direct and indirect costs due to reduced work productivity or absenteeism.^3^, ^4^, ^5^

CRS is a multifactorial disease. Mechanisms involving mucociliary clearance, epithelial barrier dysfunction, host immune response, tissue remodeling, might play a role in its pathogenesis.^6^ CRS is divided into two phenotypes: chronic rhinosinusitis with nasal polyps (CRSwNP) and without (CRSsNP). In addition, there are inflammatory endotypes of CRS that can lead to different phenotypes.^7^, ^8^ Type 1 (T1), Type 2 (T2), and Type 3 (T3) immune pathways target pathogens like viruses, parasites, and bacteria/fungi, respectively.^9^ T1 cytokines include IFN‐γ and IL‐12, T2 cytokines IL‐4, IL‐5, and IL‐13, and T3 cytokines IL‐17A and IL‐22.^9^ Each pathway is mediated by specific innate lymphoid and T‐helper cell subsets. Although overlapping inflammatory patterns exist in CRS, T2 (e.g. eosinophilic) endotypes are highly prominent in uncontrolled CRS phenotypes, as well as in CRSwNP, CRS with asthma, and nonsteroidal anti‐inflammatory drug (NSAID) exacerbated respiratory disease (NSAID exacerbated respiratory disease (N‐ERD)).^9^

The main clinical symptoms of CRS include nasal discharge, postnasal drip, nasal obstruction, hyposmia/anosmia, and facial pain.^10^ These symptoms are often subtle and non‐specific, also present in other nasal disorders, and thus the diagnosis should be confirmed by nasal endoscopy, which is the preferred method for CRS diagnosis.^2^ In addition, computed tomography (CT) scans are needed in moderate to severe cases when considering endoscopic sinus surgery (ESS) or in other situations such as with uncertain diagnosis.^2^

After failure of basic treatment including intranasal corticosteroids and nasal saline lavage, ESS is considered. CT imaging is the gold standard in the radiological evaluation of CRS before consideration of ESS.^11^, ^12^ The characteristic findings in CRS are mucosal changes within the ostiomeatal complex (OMC) and/or sinuses. Opacification of the normally aerated sinus lumen due to mucosal thickening and/or fluid content can also be found. In addition to diagnostics, CT imaging is used to evaluate the need for ESS and how extensive ESS should be performed. The most used CT staging system is the Lund‐Mackay score (LM score).^2^ It is based on the degree of sinus opacification and obstruction of OMC. LM score is a good predictor of worse outcomes after ESS.^13^, ^14^ It also correlates well with the need of baseline ESS and has a small but significant association with revision ESS.^15^

Although patient's refractory to conservative treatment often undergo ESS, trials providing high level evidence of the efficacy of ESS for CRS are missing even today.^2^, ^16^, ^17^ Patients with a prolonged and chronic disease with a severe impact on quality of life seem to benefit more from ESS than continued medical therapy.^18^ However, biologics are an alternative or adjunct therapy in severe CRSwNP cases where surgical or pharmacological options fail to provide sustained disease control and there is postoperative recurrence of NP.

Although the need for revision surgery is rather common after ESS, revision rates vary considerably in the literature partly because ESS is a highly varied procedure. According to a large prospective cohort study, the revision rate after ESS is 15% in patients with CRSsNP and 21% in patients with CRSwNP.^19^ Others have demonstrated that up to 45% of patients undergoing ESS are uncontrolled at 3 years after ESS.^20^ The number for both primary and revision surgeries was shown to be higher in CRSwNP than in CRSsNP.^21^ Choosing extensive ESS technique is usually based on the severity of mucosal inflammation and CRSwNP phenotype, yet its long‐term effect is not yet fully understood.^2^, ^22^, ^23^ Studies have shown that compared with limited ESS, extensive ESS might be superior in symptom reduction and reducing the risk of revision ESS.^23^ Moreover, patients with asthma, allergic fungal rhinosinusitis, N‐ERD, older age at first surgery, a family history of CRS, or previous sinus surgery are also more likely to require revision surgery.^24^, ^25^, ^26^, ^27^ Allergic fungal rhinosinusitis is a noninvasive form of fungal rhinosinusitis that is associated with IgE‐mediated sensitization to fungal antigens, T2 endotype, CRSwNP and living in warm, humid regions.^28^ N‐ERD is associated with the T2 endotype, asthma, CRSwNP and uncontrolled disease forms.^29^

Predictive algorithms of CRS relapses are essential in helping operation planning and decision‐making regarding biologics. Yet, there is a lack of knowledge of the predictors of CRS relapses. This multicenter study was conducted to test the utility of the Sinonasal Radiological (SR) and LM scores to predict relapses as measured by revision ESS, antibiotic courses and oral corticosteroid courses during the follow‐up.

## 2 METHODS

The study was approved by the Ethics committee of Hospital District of Helsinki and Uusimaa (Rhinosinusitis Risk Prediction, no. 31/13/03/00/2015). The study was carried out in the Departments of Otorhinolaryngology at Tampere, Kuopio and Helsinki University Hospitals, and Päijät‐Häme Central Hospital, Finland, with a waiver for written informed consent.

### 2.1 Patients

We retrospectively collected a random sample of 275 CRSwNP and 208 CRSsNP patients who had undergone sinus CT scans due to CRS between 2000 and 2018. Exclusion criteria were unavailable sinus CT scans, unavailable follow‐up data, or total LM score of CT scans <1/24. There were no patient overlaps between the current study and our previous pilot study.^1^ All patients were Caucasian.

### 2.2 Background information of the patients

The patients' background data and medical history (age, gender, smoking habits, allergic rhinitis, asthma, N‐ERD, history of systemic corticosteroid courses, duration of symptoms, endoscopic nasal polyposis, nasal polyp eosinophilia (<30% or ≥30%), and number of previous ESS) were collected from the hospital records. To determine nasal polyp eosinophilia, polyp tissue samples were processed for histological evaluation using standardized methods. The percentage of eosinophils in the total number of inflammatory cells was calculated. Asthma was doctor‐diagnosed, and the diagnosis was based on asthma drug reimbursement criteria of Finnish social security institute, in which asthma is diagnosed based on typical history and signs of reversible obstruction in lung function tests. Allergic rhinitis was doctor‐diagnosed and based on a clinical history of typical symptoms connected to specific allergen exposure and the presence of allergen‐specific IgE antibodies in serum or in skin prick test. N‐ERD was diagnosed by a positive history of airway reactions after ingestion of NSAIDs, and in 61% of N‐ERD patients, N‐ERD was additionally diagnosed by acetylsalicylic acid provocation test. The decision of the baseline and revision ESS was made by a rhinologist if CRS was uncontrolled after an appropriate medical therapy. Appropriate medical therapy was continued after ESS. Table 1 provides detailed background information. None of the patients had rare severe inflammatory conditions such as mucocele, allergic fungal rhinosinusitis, eosinophilic granulomatosis with polyangiitis, granulomatosis with polyangiitis, primary ciliary dyskinesia, or cystic fibrosis.

**TABLE 1:** Patients' background, medical history, and follow‐up characteristics.

	No ESS during late follow‐up period	ESS during late follow‐up period	*p*‐value
	*n* = 348	*n* = 135	
Male gender, *n* (%)	150 (43.1)	54 (40.0)	0.61
Age, median (Q1–Q3)	45.9 (36.8–57.4)	41.8 (31.1–50.7)	0.002*
Current smokers, *n* (%)			
No	191 (77.3)	87 (79.1)	0.78
Current	56 (22.7)	23 (20.9)	
Missing	101	25	
Allergic rhinitis, *n* (%)			
No	197 (63.8)	62 (50.0)	**0.009**
Yes	112 (36.2)	62 (50.0)	
Missing	39	11	
Asthma, *n* (%)			
No	186 (55.0)	36 (27.5)	**<0.001**
Yes	152 (45.0)	95 (72.5)	
Missing	10	4	
N‐ERD, *n* (%)			
No	259 (75.7)	60 (46.5)	**<0.001**
Yes	83 (24.3)	69 (53.5)	
Missing	6	6	
Duration of symptoms (*y*), median (Q1–Q3)	10 (3–16.5)	10 (4–17)	0.97*
≥ 1 peroral corticosteroid course/year			
No	70 (55.1)	33 (39.8)	**0.035**
Yes	57 (44.9)	50 (60.2)	
Missing	221	52	
Endoscopic NP			
No	151 (46.2)	33 (25.0)	**<0.001**
Yes	176 (53.8)	99 (75.0)	
Missing	21	3	
NP eosinophilia			
< 30%	56 (54.4)	17 (25.0)	**<0.001**
≥ 30%	47 (45.6)	51 (75.0)	
Missing of all CRSwNP cases	73	31	
Total LM score of sinus CT scans, median (Q1–Q3)	12 (6–15)	16 (12–22)	<0.001*
Total TL score of sinus CT scans, median (Q1–Q3)	2 (1–4)	4 (3–6)	<0.001*
≥ 1 previous ESS, *n* (%)			
No	221 (63.5)	65 (48.1)	**0.002**
Yes	127 (36.5)	70 (51.9)	
Baseline ESS within 1 year after the sinus CT scans			
No	57 (16.4)	18^b^ (13.3)	0.49
Yes	291 (83.6)	117 (86.7)	
Follow‐up time (y), median (Q1–Q3)	10.4 (6.3–12.0)	10.0 (7.0–12.8)	0.19*
Time until the first revision ESS (y), median (Q1–Q3)^a^	‐	2 (1–4)	‐
Revision ESS within 5 years after baseline ESS^a^			
No	272 (100)	12 (10.3)	**<0.001**
Yes	0 (0)	104 (89.7)	
Number of ESS during the follow‐up			
0	348 (100)	0 (0)	**<0.001**
1	0 (0)	106 (78.5)	
2	0 (0)	23 (17.0)	
≥ 3	0 (0)	6 (4.4)	

*Note*: *p* values by Fisher's exact test (dichotomous variables) or K Mann Whitney *U* test (continuous variables, marked with an asterisk *). Q1 = 25% percentile, Q3 = 75% percentile. Bold text indicates a statistically significant difference with a *p* value less than 0.05.

Abbreviations: ccs = corticosteroid; CRS = chronic rhinosinusitis; ESS = endoscopic sinus surgery; N‐ERD = patient‐reported NSAID‐exacerbated respiratory disease; NP = nasal polyps; Previous ESS = ESS performed before baseline computer tomography.

^a^ Only the group which underwent baseline ESS within 1 year after sinus CT scans.

^b^ Of these 18 patients, 16 had a positive history of previous CRS‐surgery/ies, and 2 did not have patient record information of a previous ESS. Missing value counts are shown for the variables that had missing values due to lack of data.

### 2.3 The follow‐up data

The follow‐up data were collected on average (min–max) 9.4 (0.2–18) years after the baseline CT scans. The data collection included baseline ESS and revision ESS during the follow‐up, prescribed and purchased courses of antibiotics due to airway infections 2016–2020, and prescribed and collected courses of peroral corticosteroids due to CRSwNP and/or asthma exacerbation 2016–2020. The search for prescription data was performed from the nation‐wide electronic prescription database. In this study, baseline ESS was defined as ESS performed up to one year after baseline CT, and revision ESS was defined as ESS later than one year after baseline CT. The time interval until ESS was calculated from the date of baseline CT scan. The decision of the baseline and revision ESS was made by a rhinologist if CRS was uncontrolled after an appropriate medical therapy. Appropriate medical therapy was continued after ESS.

### 2.4 CT scans and their evaluation

The patients underwent routine sinus multiple detector computed tomography examinations or cone beam computed tomography for clinical purposes. Two different CT machines were used: GE LightSpeed 16 (GE Healthcare, Milwaukee, Wisconsin) and Philips Brilliance 64 (Philips, Best, Netherlands). The patients were imaged in the supine position with a kilovoltage of 120 kV and a milliampere second of 100 mAs. In the GE machine, the slice thickness was 0.625 mm with coronal reconstructions at 1.5 mm. In the Philips machine, the slice thickness was 0.9 mm with coronal reconstructions at 0.9 mm. Both were three dimensional (3D) in nature without any gap. In all cases, the imaging was performed using a bone filter technique. The imaging covered the entire sinonasal area.^1^ CT scans were observed by ten experienced otorhinolaryngologists, who evaluated the SR score and the LM score for both sides of each patient on the CT scans. The otorhinolaryngologists were trained to use the SR score and LM score. The evaluators underwent training to ensure consistency, and inter‐observer agreement was achieved for the scores. We have previously studied inter‐observer and intra‐observer agreement of evaluating sinus CT scans, in which there was generally a moderate inter‐observer agreement of the structures by Cohen's kappa coefficient.^30^ In this previous study, the radiologic signs of LM and SR scores were included in this study and the average kappa that was counted of the kappa values of the structures related to SR‐score was 0.59, indicating a moderate inter‐observer agreement.^30^

#### 2.4.1 The Sinonasal Radiological (SR) score

The SR score is an advanced version of the radiological score of our previous pilot study.^1^ The SR score was formed as the total sum of 1.) non‐detectable anatomy of inferior turbinates, 0–2 points; 2.) non‐detectable anatomy of middle turbinates, 0–2 points; 3.) obstructed frontal recess, 0–2 points. Thus, the total value of SR score varies between 0 and 6 points. Non‐detectable anatomy of turbinate was defined as a situation in which the more detailed structure of the turbinate cannot be assessed. The more detailed structure of the turbinate could be for example, atrophy‐normal‐hypertrophy, pneumatization, and paradoxical turbinate. The reason for not detectable responses may be poor visualisation of the turbinate, for example, from nasal polyposis or surgical alteration of the turbinate.

#### 2.4.2 The LM score

The LM score is based on the degree of opacification (0 = normal, 1 = partial opacification, 2 = total opacification) of each sinus: maxillary, anterior ethmoid, posterior ethmoid, sphenoid, and frontal sinus, for both sides. In addition, the OMC is graded as 0 = not occluded, or 2 = occluded, coming to a maximum score of 12 per side.^15^, ^31^

### 2.5 Data analysis

**FIGURE 1:**
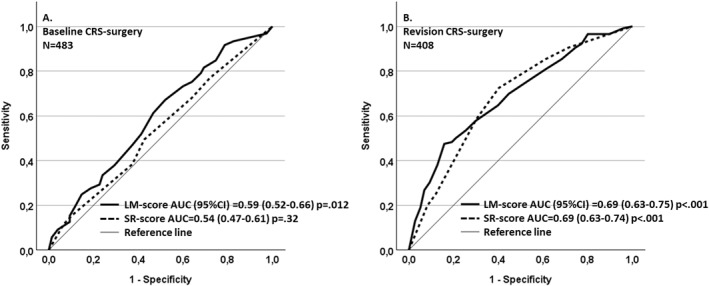
Predicting probability of the two radiological scores of sinus computer tomography (CT) scans of chronic rhinosinusitis (CRS) patients. The scores were Sinonasal Radiological (SR) score (a total of 0–6 points) and Lund‐Mackay (LM) score (a total of 0–24 points). (A) All 483 subjects. Both scores poorly identify the patient group undergoing endoscopic sinus surgery (ESS) at the baseline. (B) Only 408 subjects underwent baseline ESS within one year after baseline CT. Both scores significantly identify revision ESS. With the threshold value LM score ≥ 13/24, the sensitivity was 65% and specificity 61% for detecting those who needed revision ESS during follow‐up after the baseline ESS. With the threshold value SR score ≥ 2/6, the sensitivity was 72% and specificity 60% for detecting those who needed revision ESS during follow‐up after the baseline ESS. The threshold values were counted by the coordinates of the ROC curve (data not shown). AUC, area under curve;AUROC, area under the receiver operating characteristic curve; CI, confidence interval.

Statistical analysis was carried out using the Statistical Product and Service Solutions (SPSS) Base 15.0 Statistical Software Package (SPSS Inc., Chicago, IL, USA). The baseline ESS rate, the follow‐up revision ESS rate, antibiotic courses and peroral corticosteroid courses during the follow‐up were signs of interest. Associations were assessed by nonparametric tests: Fisher's exact test (to evaluate associations between dichotomous variables), and Mann Whitney U tests (to compare two independent groups regarding continuous variables, SR or LM scores). Area under the receiver operating characteristic curve (AUROC) curve was used to evaluate the predictive potential. AUROC is a performance metric that is used to evaluate classification models (SR score or LM score). In the models, the events were “baseline ESS” or “revision ESS”. The AUROC gives the probability that a randomly selected patient who experienced an event will have a higher predicted risk score than a randomly selected patient who did not experience an event. The cut‐off values for the LM and SR scores were assessed by using the data of coordinates of the AUROC (Figure 1). The predictive potential of SR and LM scores over time was assessed by plotting the Kaplan‐Meier survival curves. Differences between groups were evaluated using the log‐rank test, which compares the survival distributions across groups to estimate hazard ratios (HRs) for revision ESS rates. Multivariable Cox's proportional hazards models were used to evaluate the HRs of revision ESS rates of the following variables: LM‐score, SR‐score, previous ESS, baseline NP, asthma and/or N‐ERD (asthma/N‐ERD). This method assesses the impact of predictor variables on the time until an event (revision ESS). CRSwNP, previous ESS and asthma/N‐ERD variables had missing values, and they were regarded as “no” in the models. Most variables (such as LM‐, SR‐scores, age, gender, baseline ESS, revision ESS, time until the revision ESS, follow‐up time) were not having missing values. Overall, the numbers of missing values are shown in Table 1. To test the proportional hazard assumption of the Cox's proportional hazards model, we included time‐dependent covariates. For most variables (LM‐score, asthma/N‐ERD and previous ESS) time‐dependent covariates were not significant; hence, most predictors satisfied the proportional hazard assumption. To assess multicollinearity among the variables, Variance Inflation Factors were computed for each variable as well as correlation coefficients were calculated for all variable pairs. The relationship between two continuous variables was evaluated using the Spearman rank correlation test, between continuous and binary variables was evaluated using the Point‐Biserial Correlation, while the relationship between binary variables was examined using the Phi Coefficient. All VIFs were below 2 and correlation coefficients (|*r*|) were less than 0.5 for the pairwise correlations, except for the correlation between SR‐score and LM‐score (*r* = 0.79) and between baseline NP and LM‐score (*r* = 0.58). Due to potential collinearity and limited satisfaction of the proportional hazard assumptions, sensitivity analysis was performed in multivariable Cox's model by omitting LM‐score, SR‐score, baseline NP, SR‐score + baseline NP, and LM‐score + baseline NP. All statistical tests were two‐tailed, and *p*‐values < 0.05 were considered statistically significant. Results for continuous variables are presented as medians with 25th (Q1) and 75^th^ (Q3) percentiles.

## 3 RESULTS

### 3.1 Patient characteristics

The patient characteristics are shown in Table 1. For analysis, patients were divided into two groups: 1) patients who underwent ESS during the late follow‐up period (late ESS group) and 2) patients who did not undergo ESS during the late follow‐up period (no late ESS group). The late follow‐up period here meant the period starting one year after the baseline CT. The median age was significantly lower, and the tissue eosinophilia was significantly higher in the late ESS group. The proportion of patients with baseline peroral corticosteroid course, NP, asthma, AR, N‐ERD, and a positive history of a previous ESS was significantly higher in the late ESS group. Other background, medical history, or follow‐up factors did not differ between the groups. All the above‐mentioned data with *p*‐values ​​are presented in detail in Table 1.

### 3.2 SR and LM score and baseline ESS

Baseline ESS, that is ESS performed within one year of the baseline CT, was performed in 408 (84.5%) patients. The median (Q1–Q3) SR score was 2^1^, ^2^, ^3^, ^4^ in the baseline ESS group, and it was 2 (0–4) in the non‐baseline ESS group (*p* = 0.16). The median (Q1‐Q3) LM score was 13^9^, ^10^, ^11^, ^12^, ^13^, ^14^, ^15^, ^16^, ^17^, ^18^ in the baseline ESS group, and it was 11^5^, ^6^, ^7^, ^8^, ^9^, ^10^, ^11^, ^12^, ^13^, ^14^, ^15^ in the non‐baseline ESS group (*p* = 0.011). The AUROC values were 0.54 (SR score) and 0.59 (LM score), indicating poor predictive potential of baseline ESS performed within one year after the sinus CT scans (Figure 1A). SR and LM scores correlated significantly (*p* < 0.01, *r*_*S*_ = 0.79).

### 3.3 SR score and LM score predict ESS during follow‐up

**FIGURE 2:**
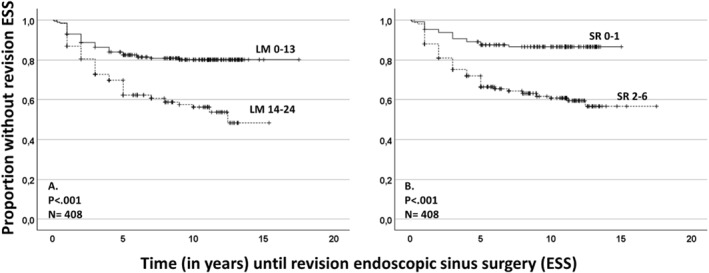
Kaplan–Meier survival curves showing how two scores of computed tomography scans taken before the baseline surgery are able to predict the risk of revision endoscopic sinus surgery (ESS). The two radiological scores were Sinonasal Radiological (SR) score (a total of 0–6 points) and Lund‐Mackay (LM) score (a total of 0–24 points). The patient group (408 patients) who had undergone baseline ESS within one year after the baseline CT was observed. Baseline = ESS performed within one year from the date of CT scan; Revision = ESS performed over a year after the date of the CT scan; *p*‐values by log rank test.

Revision ESS was performed in 133/483 (28.0%) patients on average (min–max) 3.2 (0–12) years after the time of performing the baseline sinus CT scans. Two patients in the late ESS group (135 patients) had no prior ESS (previous ESS or baseline ESS) (Table 1). When observing only the group that underwent baseline ESS within one year after performing the sinus CT scans (408 patients), the AUROC values were 0.68 (SR score) and 0.69 (LM score), indicating good predictive potential of revision ESS (Figure 1B). The patients with high baseline SR score or LM score had statistically significantly higher revision ESS‐rate compared with those with low scores (*p* < 0.001, Figure 2).

Among patients who underwent baseline ESS, we performed univariate Cox's hazard models to evaluate radiological and clinical factors predicting revision ESS. When compared to non‐revised patients, revision ESS was significantly associated with the following variables; LM_14–24_ value (HR 2.47, 95% confidence interval [CI] 1.69–3.62, *p* < 0.001); SR_2–6_ value (HR 3.35, 2.00–5.61, *p* < 0.001); a history of a previous ESS before baseline ESS (HR 1.68, 1.16–2.41, *p* = 0.006); baseline endoscopic signs of NP (HR 2.67, 1.73–4.14, *p* < 0.001) and presence of comorbid asthma and/or N‐ERD (HR 2.91, 1.95–4.33, *p* < 0.001). When all these five variables were added to the same multivariable model, only the following two variables were significantly associated with the revision ESS; SR_2–6_ value (HR 1.95, 1.05–3.62, *p* = 0.035); and asthma/N‐ERD (HR 1.89, 1.19–3.00, *p* = 0.007).

Sensitivity analysis was performed in multivariable Cox's model by omitting LM‐score, SR‐score, baseline NP, SR‐score+baseline NP, and LM‐score + baseline NP. When omitting LM‐score in this multivariable model, the result remained similar: the following two variables were significantly associated with the revision ESS; SR_2–6_ value (HR 2.05, 1.14–3.67, *p* = 0.016); and asthma/N‐ERD (HR 1.93, 1.22–3.04, *p* = 0.005). When omitting SR‐score, the result remained similar: asthma/N‐ERD was significantly associated (HR 2.07, 1.31–3.28, *p* = 0.002). When omitting baseline NP, the result remained similar: the following two variables were significantly associated with the revision ESS; SR_2–6_ value (HR 2.11, 1.15–3.85, *p* = 0.016); and asthma/N‐ERD (HR 1.97, 1.25–3.11, *p* = 0.004). When omitting both baseline NP and LM‐score, the result remained similar: the following two variables were significantly associated with the revision ESS; SR_2–6_ value (HR 2.37, 1.37–4.11, *p* = 0.002); and asthma/N‐ERD (HR 2.01, 1.34–3.25, *p* = 0.001). When omitting both SR‐score and baseline endoscopic signs of NP, the result remained similar: asthma/N‐ERD was significantly associated (HR 2.20, 1.39–3.47, *p* < 0.001).

## 4 ANTIBIOTIC/PERORAL CORTICOSTEROID COURSES DURING THE FOLLOW‐UP

The data of peroral corticosteroid/antibiotic courses purchased during the years 2016–20 were available from 187 patients. The median (Q1–Q3) min‐max number of peroral corticosteroid courses/year in SR_2–6_ group was 0.5 (0–1) 0–4.5, and in the SR_0–1_ group it was 0 (0–0) 0–3 (*p* = 0.007). The difference remained statistically significant when observing only the group who had undergone baseline ESS (*p* = 0.009), whereas the result was insignificant when observing only the group who had not undergone baseline ESS (*p* = 0.33). The median (Q1–Q3) min–max number of peroral corticosteroid courses/year in LM_14–24_ group was 0.5 (0–1) 0–4.5, and in LM_0–12_ group it was 0 (0–0.5) 0–3 (*p* < 0.001). The difference remained statistically significant when observing only the group who had undergone baseline ESS (*p* < 0.001), whereas the result was insignificant when observing only the group who had not undergone baseline ESS (*p* = 0.74).

The number of antibiotic courses purchased/year during the follow‐up did not differ significantly between the SR_2–6_ and SR_0–1_ groups (*p* = 0.064). The results remained similar when stratifying by baseline ESS or revision ESS. The number of antibiotic courses purchased/year during the follow‐up did not differ significantly between the LM_high_ and LM_low_ groups (*p* = 0.24, by MWU test). The results remained similar when stratifying by baseline ESS or revision ESS.

## 5 DISCUSSION

Predictive algorithms of CRS relapses are essential in helping operation planning and decision‐making regarding biologics. This study aimed to evaluate the predictive potential of the following variables, SR‐, LM‐scores and patient history factors, on CRS relapses. Our main finding was that both SR and LM scores predicted revision ESS and the need for oral corticosteroids (OCS) courses during the follow‐up. In the multivariable model, however, SR score and asthma/N‐ERD predicted revision ESS, whereas LM score, a history of a previous ESS before baseline ESS and CRSwNP phenotype were not associated with revision ESS risk. The need for revision of ESS and/or OCS reflects CRS relapse or uncontrolled diseases. The revision ESS is a treatment option when the medical therapy fails to achieve a balance of care in CRS.^2^ The most appropriate treatment for each patient should be selected individually. The main goal of our present study was to identify early predictive markers from sinus CT scans to predict which patients have an increased risk of uncontrolled CRS in the follow‐up.

We have previously shown in a pilot study that a radiological score of sinus CT scans can predict the need for revision ESS, thus putatively predicting cases that remain uncontrolled despite treatment.^1^ This multi‐center study aimed to develop a scoring system and to validate the predictive potential of the SR score in a random sample of CRS patients that were different from those in the pilot population.

The number of antibiotic courses purchased/year and the number of peroral corticosteroid courses purchased/year during the follow‐up were also used as a sign of recalcitrant CRS in the present study. We found that SR score or LM score did not predict the need for antibiotic courses during follow‐up. Instead, a high LM score and a high SR score predicted a higher number of peroral corticosteroid courses during follow‐up. Peroral corticosteroid courses are more commonly used to treat uncontrolled CRSwNP than recalcitrant CRSsNP. Our findings indicate that both SR and LM scores may detect uncontrolled CRSwNP patients.

Early detection of CRS relapses is essential in the planning of operation and decision making of advanced therapeutics such as biologics. We also found that the CRSwNP phenotype and the presence of asthma and/or N‐ERD strongly predicted revision ESS. The present study and previous observations have shown that the risk of revision ESS was increased in patients with baseline lower median age, higher tissue eosinophilia, peroral corticosteroid course(s), CRSwNP phenotype, comorbid asthma, AR, N‐ERD, and a positive history of previous ESS.^24^, ^25^, ^27^, ^32^, ^33^, ^34^ Hence, it would be useful in the future to study the predictive potential of a combination of radiological scoring system and other parameters in a real‐life setting.^35^ A prospective study from the UK demonstrated a small but statistically significant association between LM score and revision rates at 36 months.^15^

This study uses the need for revision ESS as a marker of disease recurrence in CRS patients. However, it is important to contextualize this marker within the broader clinical management of CRS. Biologic therapy, increasingly utilized in CRS with NP (CRSwNP), is typically indicated for patients with recurrent NP and uncontrolled disease despite surgery and medical therapy.^36^

Extent of ESS was chosen based on disease severity, extent of inflammation and presence of CRSwNP phenotype. A systematic review and meta‐analysis has shown that compared to limited ESS with least tissue removal or ESS not addressing all sinuses, a full‐house ESS as complete bilateral all sinuses opening with large middle meatal antrostomies and Draf 2a frontal sinusotomy might be superior in symptom reduction and reducing the risk of revision ESS.^23^ These findings suggest that surgical technique alone may not fully determine the duration of disease control. There is still an unmet need to study which ESS technique should be used in which patient and the long‐term effect of each ESS technique on the symptom reduction and risk of revision ESS.^2^

There are several limitations of our study that need discussion. Data was unfortunately not available on the extent of ESS, and hence, this needs to be explored in the future. We acknowledge that postoperative CT scans were not available due to radiation hygiene reasons. These reasons also limit the routine use of SR scores in all CRS patients. On the other hand, SR scoring can show how CT image data could be used more efficiently when CTs need to be performed. We acknowledge that preoperative CT scans cannot account for inter‐surgeon variability in surgical techniques, which is a common challenge in multicenter studies. This variability might influence disease recurrence and the need for revision of ESS. Moreover, data of inter‐observer agreement were not available. However, the evaluators underwent training to ensure consistency, and we have previously demonstrated moderate (fair to good) inter‐observer agreement of these signs of the sinus CT scans.^24^ In this previous study, poor reproducibility was observed in the following structures: optic nerve, insertion of the uncinated process, anterior ethmoidal artery, and Keros class, which we did not evaluate in the current study. We acknowledge that data and variables were lacking from important variables such as eosinophil count, symptom and olfaction scores, and use of biological therapy due to the retrospective nature of this study. However, biological therapy was not available for CRSwNP during the time the study was conducted. It might be possible in some cases that revision ESS may have been influenced by a number of factors unrelated to the actual need for revision ESS, including waiting times for the surgery and the individual needs of the patient to delay surgery, and by factors such as patient's tolerance of CRS symptoms, the extent of primary ESS, doctor's, and patient's personal opinions, and seeking treatment elsewhere. However, revision ESS has been shown to reflect uncontrolled CRS.^1^ Due to the homogeneity of the patient material (all were Caucasian), there may be limitations in generalizing the results to other populations. In the future, a study using artificial intelligence would be useful to see whether it captures similar scoring systems and findings in the corresponding research question. Indications for ESS extend beyond disease recurrence and include specific cases such as mucoceles, fungal sinusitis, and certain orbital or intracranial complications. These distinct indications could impact the study cohort if present. As our data had no cases with these diseases, our findings of the predictive role of the CT score cannot be extrapolated to the rare conditions. Overall, despite the challenges of our data, preoperative CT scoring might be a useful tool for predicting CRS outcomes.

## 6 CONCLUSION

LM and SR, as well as clinical background information such as history of asthma/N‐ERD, may be useful in predicting CRS relapses, which may help in operation planning or decision‐making for biologics. Yet, more studies in other populations are needed.

## AUTHOR CONTRIBUTIONS

All authors helped shape the research and data collection, participated in writing and provided critical feedback on the manuscript. S.T.‐S. and H.H. performed statistical analyses.

## CONFLICT OF INTEREST STATEMENT

STS reports consultancies for ALK‐Abelló, AstraZeneca, Clario, ERT, GlaxoSmithKline, Novartis, Sanofi Pharma, Orion Pharma, Roche Products and grants from GlaxoSmithKline and Sanofi. All are outside the submitted work. All other authors declare no conflicts of interest.

## CONSENT

Ethics approval for the study (number 31/13/03/00/2015) was granted by the relevant Hospital Districts' ethics committee, with a waiver for written informed consent.

## Data Availability

Due to Finnish data protection legislation and confidential health‐related data, the datasets produced and/or examined during this study are not accessible to the general public. They can solely be managed by designated individuals within the study group for specific research objectives. The datasets analyzed during the current study are available from the corresponding author upon reasonable request. Data use permissions can be applied from the competent authorities.
